# Employing Parasite Against Cancer: A Lesson From the Canine Tapeworm *Echinococcus Granulocus*

**DOI:** 10.3389/fphar.2019.01137

**Published:** 2019-09-25

**Authors:** Wang Guan, Xiaoqin Zhang, Xiao Wang, Shun Lu, Jun Yin, Jinxin Zhang

**Affiliations:** ^1^Department of Cancer Prevention and Treatment, Sichuan Cancer Hospital and Institute, Sichuan Cancer Center, School of Medicine, University of Electronic Science and Technology of China, Chengdu, China; ^2^Intensive Care Unit, Sichuan Provincial People’s Hospital, School of Medicine, University of Electronic Science and Technology of China, Chengdu, China; ^3^Radiation Oncology Key Laboratory of Sichuan Province, Sichuan Cancer Hospital and Institute, Sichuan Cancer Center, School of Medicine, University of Electronic Science and Technology of China, Chengdu, China; ^4^School of Public Health, Sun Yat-sen University, Guangzhou, China

**Keywords:** *Echinococcus granulosus*, cancer, immunotherapy, immune response, adjuvant

## Abstract

Cystic echinococcosis (CE), a devastating zoonotic condition caused by the tapeworm *Echinococcus granulosus*, remain a significant public health problem worldwide. However, after a negative correlation between solid tumor and CE has been incidentally discovered, accumulating evidence have suggested that this parasite may induce anticancer effect through activating host immune response and secreting molecules with anticancer potential, which may provide some new understanding for immunotherapy. This article will review the evidence supporting the anticancer effect of *E. granulosus* and its underlying mechanisms and discuss the possible implications in immunotherapy.

## Introduction

With a new era of immuno-oncology on the horizon, numerous cancer patients have benefited from the great advances in immunotherapy, especially the discovery of immune checkpoint inhibitors (ICIs), such as cytotoxic T lymphocyte-associated protein 4, programmed cell death 1, and programmed cell death ligand 1 inhibitors (Darvin et al., 2018). However, the great advantages of these immunotherapeutic strategies are also hampered by major limitations, which restrict them from benefiting a broader range of cancer patients. For example, only 20% of patients with advanced carcinoma (except Hodgkin’s lymphoma and melanoma) would benefit from programmed cell death 1 or programmed cell death ligand 1 inhibitors (Balar and Weber, 2017). Similarly, the clinical response rate is very low even in patients receiving anti-CTLA4 therapy for unresectable or metastatic melanoma (the first approved indication of anti-CTLA4 therapy) (Savoia et al., 2016). Meanwhile, patients receiving ICIs, whether in single drug or in combination, would experience unique immune-related adverse events, ranging from mild adverse effect to potentially life-threatening events (Champiat et al., 2015). Hence, immunotherapeutic strategies employing other potential mechanisms are worthy of further exploring.

*Echinococcus granulosus*, a canine tapeworm responsible for zoonotic cystic echinococcosis (CE; also termed hydatid disease), has haunted human kind for centuries (Wen et al., 2019). *E. granulosus* is still highly epidemic in South America, Mediterranean countries, eastern Africa, Central Asia, and Northwestern China, with human incidence as high as 50 *per* 100,000 person-years according to a report from the World Health Organization (Brunetti et al., 2010). However, after the astonishingly low incidence of CE was incidentally found in patients with solid tumor in Turkey (Akgül et al., 2003), accumulating evidence have suggested that *E. granulosus* may exhibit anticancer effect through host immune system, which may be employed as an immunotherapeutic strategy against cancer (Ranasinghe and McManus, 2018).

### *E. Granulosus* Against Cancer: Incidental Findings to Accumulating Evidences

Various helminths have been proven to be a carcinogenic agent in human, such as the liver fluke *Clonorchis sinensis* and *Opisthorchis viverini* (causative agents of cholangiocarcinoma) (Feng and Cheng, 2017) and the blood fluke *Schistosoma japonicum* (well-known risk factor for liver carcinoma and colorectal cancer) (Takemura et al., 1998; Hamid, 2019), *Schistosoma mansoni* (suspected risk factor for colorectal cancer) (Almeida et al., 2017), and *Schistosoma haematobium* (which leads to bladder cancer) (Berry et al., 2017). On the other hand, accumulating evidence suggested that certain helminth infection could induce anticancer activities, such as the pork worm *Trichinella spiralis*, which can protect infected mice against tumor growth and metastasis (Kang et al., 2013).

Coexistence of *E. granulosus* infection and cancer has been broadly reported, such as coexistence with hepatocellular carcinoma (Bourne and Williams, 1963; Zöld et al., 2005; Li et al., 2015), lung carcinosarcoma (Misthos et al., 2012), liver mucinous cystadenoma (Muralidhar et al., 2018), renal sarcoma (Benchekroun et al., 1979), renal adenocarcinoma (Miñana López et al., 1994), or ovarian epithelial tumor and lymphoepithelioma-like gastric carcinoma (Gungor et al., 2011). The exact relationship between *E. granulosus* and cancer, however, has long been unclear until the last decade, when an epidemiological study on patients with CE occasionally found a negative correlation between CE and solid tumors (Akgül et al., 2003). This extraordinary phenomenon, although still in debate (Oikonomopoulou et al., 2016), has led to a hypothesis that infection of *E. granulosus* may elicit protective effect against cancer. Subsequently, results from studies carried out by different research groups have supported this hypothesis. It has been demonstrated that, for example, protoscolices in hydatid cyst (the larval stage of *E. granulosus*) could induce cell death in WEHI-164 fibrosarcoma cell *in vitro* (Darani et al., 2012). Moreover, vaccination with hydatid fluid induced tumor regression in a mouse model with experimental CT26 colon cancer (Berriel et al., 2013). In addition, simultaneously injection of alive protoscolices and melanoma cell could result in significantly reduced tumor growth compared with control group in a mouse model (Chookami et al., 2014). Similarly, intraperitoneal hydatidosis established by infection of protoscolices could inhibit DMBA-induced mammary tumorigenesis in rats (Altun et al., 2015). As an indirect evidence, sera from patients with hydatid disease was cytotoxic to lung cancer cell line but had no effect on fibroblast cells (Karadayi et al., 2013). Collectively, these evidences suggested that *E. granulosus* may elicit protective effect against certain cancer types *in vitro* and *in vivo*.

### Mechanisms Underlying Effect Induced by *E. Granulosus*

**Figure 1:**
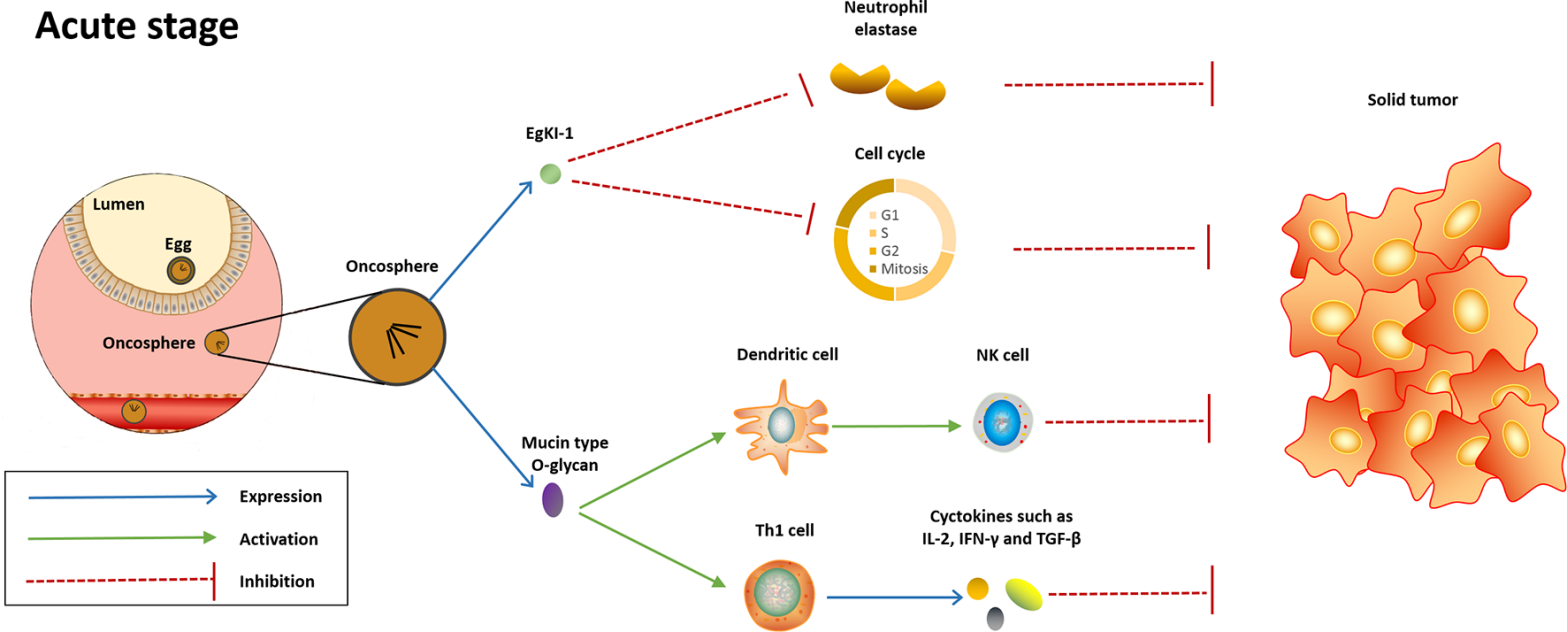
Potential mechanisms underlying the anti-cancer effect induced in the acute stage of *Echinococcus granulosus* infection. In the acute stage, EgKI-1 release by oncosphere will potently null the neutrophil elastase and disrupt cell cycle, therefore inducing anticancer effect against several cancer types. Meanwhile, the mucin-type O-glycan of this parasite will be recognized by host immune system, thus activating innate and Th-1-polarized immune responses, which are protective against cancer.

**Figure 2:**
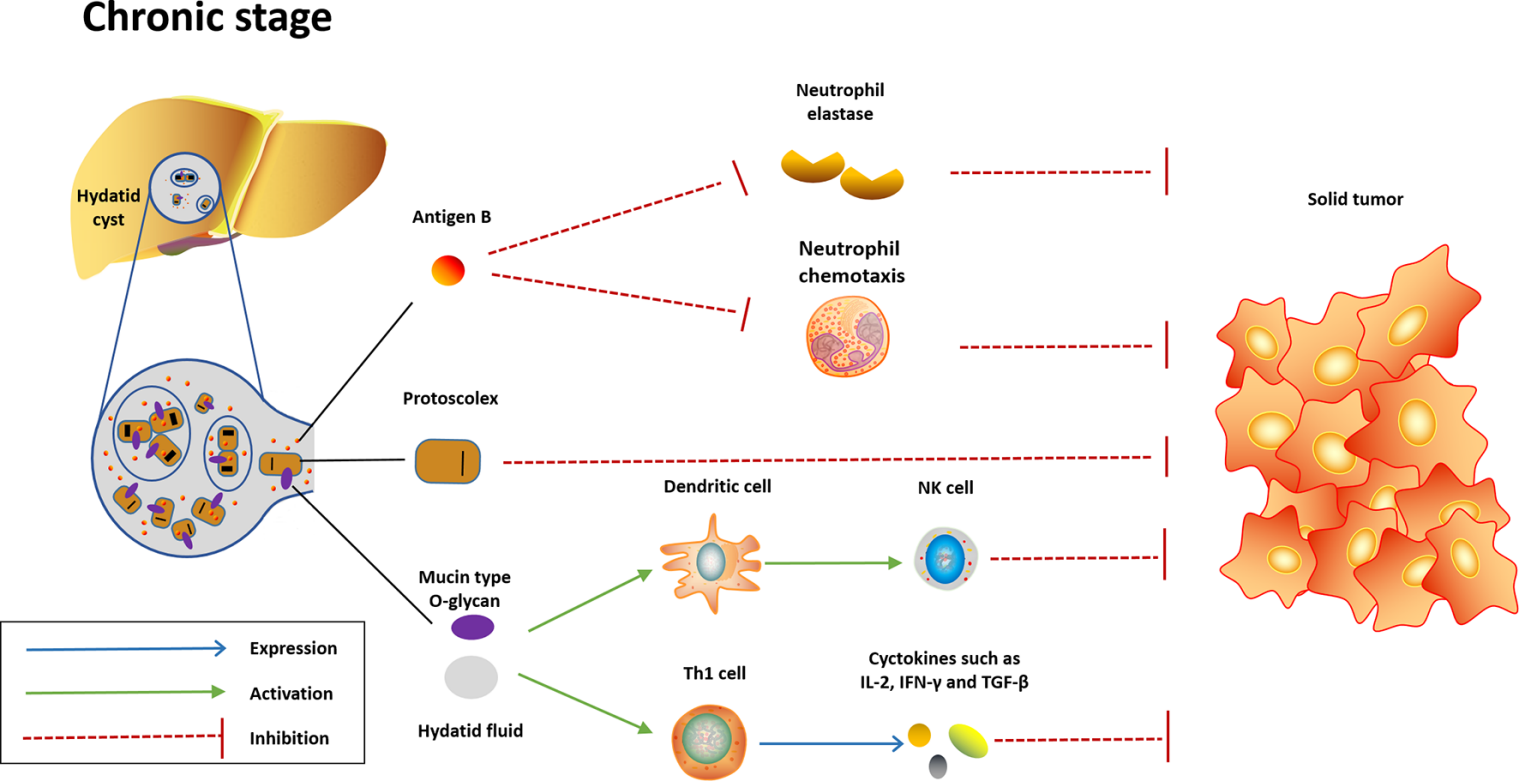
Potential mechanisms underlying the anticancer effect induced in the chronic stage of *Echinococcus granulosus* infection. In the chronic stage, the content of hydatid cyst will be released into liver or other infection sites when the cyst dies or ruptures. Highly immunogenic content of hydatid cyst will quickly activate innate immunity and convert Th-2 response to Th-1 response, which are protective against cancer. AgB, another potent neutrophil elastase inhibitor highly expressed in the hydatid cyst, may exhibit anticancer effect through inhibition of neutrophil elastase and neutrophil chemotaxis. Meanwhile, the protoscolex may also play a role in the anticancer effect.

Despite the great efforts put by researchers, little is known about the mechanisms underlying the anticancer effect induced by *E. granulosus*. Several potential mechanisms have been proposed, including direct anticancer effect induced by parasite molecules and indirect anticancer effect through activation of host immune response (**Figures 1** and **2**).

### Direct Anticancer Effect Induced by Parasite Molecules

Several studies have suggested that *E. granulosus* could kill cancer cell or inhibit tumor growth. As noted above, protoscolex could induce cell death in WEHI-164 fibrosarcoma cell *in vitro* (Darani et al., 2012) and inhibit melanoma tumor growth *in vivo* (Chookami et al., 2014). The specific molecules involved in this process, however, remain largely unclear. Notably, a study found that treatment with different hydatid molecules, especially the protoscolex excretory/secretory (ES) molecules, could increase the number of dead cells and/or decrease the number of alive cells in HeLa cell culture; meanwhile, treatment with the same molecules neither increase the number of dead cells nor decrease the number of alive cells in vero cell culture (Aref et al., 2013). Subsequently, another study found that injection of hydatid fluid (peritoneal or tumor margin) could decrease melanoma tumor size in C57 mouse model (Darani et al., 2016). However, the alum-based adjuvant employed in the latter study is potentially confounding (Ranasinghe and McManus, 2018), since alum may induce anticancer effect through selectively stimulation of Th2 immune response (Didierlaurent et al., 2009). Recently, a research group found that the Kunitz-type protease inhibitor EgKI-1, a potent chymotrypsin and neutrophil elastase inhibitor highly expressed by oncosphere of *E. granulosus* (Ranasinghe et al., 2015), could inhibit several human cancers from growth and migration, probably through disrupting cell cycle and inducing apoptosis of cancer cells, without affecting normal cell growth *in vitro* (Ranasinghe et al., 2018). Meanwhile, EgKI-1 treatment could restrict experimental triple negative breast cancer growth in a BABL/c mouse model. The specific mechanism underlying this effect still demands further exploring. A possible explanation is that EgKI-1 may decrease cancer cell migration *via* potent inhibition of neutrophil elastase, which plays a pivotal role in cancer metastasis (Zhang et al., 2016). Being another potent neutrophil elastase inhibitor highly expressed in the hydatid cyst, it has been hypothesized that Antigen B (AgB) may be responsible for the anticancer effect induced during chronic state of infection. Recently, another study explored the anticancer potential of several hydatid fluid antigens, including AgB, glycolipid, glycoprotein, and 78 kDa fractions in breast cancer cell lines. Compared with the other molecules, less apoptosis was observed after treatment with AgB fraction (Daneshpour et al., 2019). Therefore, the possible role of AgB requires further exploring. In general, available evidence suggested that the molecules from the hydatid cyst, especially the ES molecules, are potentially responsible for the anticancer effect of this parasite.

### Indirect Anticancer Effect Through Activation of Host Immune System

As noted above, anticancer effect has been seen in various parasites infection. Similar with parasites infection, cancer cells could also trigger innate and adaptive immunity in the process of development (Trinchieri, 2015). Therefore, it has been hypothesized that parasites may induce indirect anticancer activities *via* boosting host immune system when chronically infected at a low density (Darani and Yousefi, 2012), i.e., parasites infection may play as negative regulator of cancer through reactivation of host immune system (Callejas et al., 2018).

In favor of this hypothesis, antigen similarity between several parasites and certain cancer types has been reported, especially the cancer-associated mucin-type *O*-glycans (Osinaga, 2007), thus allowing crosstalk between parasites and carcinomas to ensue. For example, *S. mansoni* can express the Tn antigen (Nyame et al., 1987), which is associated with many cancer types (including bladder, cervix, colon, ovary, gastric, lung, and prostate cancer) and plays an important role in cancer metastasis and evasion from immunosurveillance (Fu et al., 2016). Meanwhile, the Tk antigen, which is strongly expressed at the surface of a significant proportion of human colorectal carcinomas (Meichenin et al., 2000), has been detected in *Taenia hydatigena*, *Taenia crassiceps*, and *Mesocestoides vogae* (Ubillos et al., 2007). In the case of *E. granulosus*, the initial evidence of common antigen shared with cancers came from a report published in late 1970s, which found that an immunoelectrophoresis test with hydatid fluid and serum from a patient with pulmonary carcinoma resulted in an intense precipitin band (Yong et al., 1979). Thereafter, different research groups have independently reported the antigen similarity between *E. granulosus* and several cancer types, especially the mucin-type O-glycans. As an example, cancer-associated Tn antigen and Sialyl-Tn (sTn) antigen have been detected in both larva and adult worm extracts and in sera from patients with hydatid disease (Alvarez Errico et al., 2001). In addition, TF antigen has been characterized in adult worm (Casaravilla et al., 2003). Besides, common antigens other than mucin-type *O*-glycans have also been detected. For example, a nonglycosylated 27 kDa molecule was shared by human breast cancer and *E. granulosus* (Sharafi et al., 2016). Similarly, the hot shock protein 70 of *E. granulosus* displayed 60% homology with the mortalin of CT26 colon cancer cell (Berriel et al., 2013). Moreover, it has been demonstrated that the common antigens shared by E. granulosus and cancers could induce immunological cross-reaction. For example, it has been demonstrated that antigens from hydatid cyst could react with sera from patients with breast cancer, while antisera raised against laminated and germinal layers of hydatid cyst could react with ES products of cancer cell (Daneshpour et al., 2016). In addition, a 40-kDa antigen from hydatid fluid could react with sera from patients with breast cancer, while monoclonal antibody raised against this 40-kDa antigen could react with breast cancer cell (but cell growth was unaffected) (Sharafi et al., 2017). Altogether, it is reasonable to hypothesize that *E. granulosus* may exhibit anticancer effect through adaptive immunity induced by common antigens.

Previous studies have demonstrated that both cellular immunity and humoral immunity induced by protozoan parasite *Trypanosoma cruzi* are protective against several cancer types in mice model (Kallinikova et al., 2008; Zenina et al., 2008; Zhigunova et al., 2013). In the case of *E. granulosus*, however, it is yet unclear which kind of immunity is more important in the anticancer effect. In the paradigm of onco-immunology, it has been recognized that Th1-polarized response plays pivotal role in killing cancer cell and inhibiting tumor growth, whereas Th2-polarized response promotes tumor progression and metastasis (Shurin et al., 1999; Zamarron and Chen, 2011). As for *E. granulosus*, the immunity induced by parasite varies at different infection stages: 1) During oncosphere invasion, a Th1-polarized response will dominate; 2) in the process of cyst formation and growth, a Th2-polarized response will gradually take in charge; 3) when cyst ruptures or dies, the Th2-polarized response will be rapidly taken over by Th1-polarized response (Zhang et al., 2008; Gottstein et al., 2017). Therefore, it has been hypothesized that the anticancer effect may come from the Th1-polarized response induced at specific stages of infection (Tez and Tez, 2015). In addition, this may provide an explanation for the contradictive results from some epidemiological investigation (Oikonomopoulou et al., 2016) and laboratory research (Turhan et al., 2015), which showed that CE may promote cancer development and progression.

There are some evidence that support the role of humoral immunity in the anticancer effect induced by *E. granulosus*. As noted above, sera from patients with hydatid disease was cytotoxic to lung cancer cell line but had no effect on fibroblast cells (Karadayi et al., 2013). However, the humoral immunity seems unimportant in the anticancer effect induced by *E. granulosus* in subsequent studies. As an example, antibody against Tn-like peptide from *E. granulosus* scarcely reacted with mammary adenocarcinoma cell line TA3/Ha and pancreatic adenocarcinoma cell line Panc02 (both of which strongly express Tn antigen), despite the peptide induced high level of antibody production; meanwhile, high level of interferon gamma (IFN-γ) was detected in mice immunized with this peptide, while interleukin (IL)-5 and IL-17 were not detected, indicating a Th1-dominant response (Noya et al., 2013). Similarly, immunization of mice bearing melanoma cancer with hydatid cyst antigens resulted in inhibition of tumor growth and IFN-γ production (Rostami et al., 2018). Moreover, the monoclonal antibody raised against the 40-kDa antigen hydatid fluid mentioned above could not restrict breast cancer cell growth *in vitro*, even though it could react with breast cancer cell (Sharafi et al., 2017). Recently, a study found that passive transfer of antisera against hydatid fluid, protoscolex antigen, or cyst wall antigen did not affect melanoma tumor growth in recipient mice that had already been challenged by melanoma cells (Darani et al., 2018). On the other hand, passive transfer of spleen cells from mice immunized with hydatid cyst, hydatid fluid, or protoscolex to recipient mice would lead to significant reduction in melanoma tumor size and tumor growth rate (Ramazninia et al., 2016; Darani et al., 2018). Therefore, Th1-polarized response are more likely to be responsible for the anticancer effect induced by *E. granulosus*.

Besides adaptive immunity, the study noted above also detected high level of natural killer cell activation, thus indicating a role of innate immunity in the anticancer effect (Noya et al., 2013). However, a definite conclusion cannot be reached in the current stage due to lack of evidence.

In summary, *E. granulosus* may induce anticancer effect through direct anticancer effect, i.e., secreting various molecules with cancer killing effect, and indirect anticancer effect, i.e. activating host immune response (Th1-polarized response in particular). However, more efforts are required to shed further light on the mechanisms underlying this extraordinary effect.

### Implication in Cancer Immunotherapy

In the long-lasting campaign against cancer, four general approaches have been developed: surgery, chemotherapy, radiotherapy, and most recently, immunotherapy, which has evolved into a promising cancer treatment modality (Floudas et al., 2019). Inspired by the victory achieved with ICIs in the last decade, oncologists and immunologists have made every endeavor to secure further success. However, the success achieved with ICIs is still limited, thus demanding more directions to be explored. The incidental discovery of anticancer effect induced by *E. granulosus* infection have gained much focus. Understanding the underling mechanisms may provide some new insights for immunotherapy orchestration.

### Echinococcal Molecules With Anticancer Potential

Available evidences have led to a hypothesis that *E. granulosus* may induce anticancer effect through hydatid molecules, especially the protoscolex ES molecules. Although the specific molecules and the underlying mechanisms remain largely unknown, EgKI-1, a potent chymotrypsin and neutrophil elastase inhibitor highly expressed by *E. granulosus* (Ranasinghe et al., 2015), was able to induce anticancer effect both *in vitro* and in animal models (Ranasinghe et al., 2018). In addition to the potent inhibitory effect on neutrophil elastase, which plays a crucial role in cancer metastasis, EgKI-1 can also induce anticancer effect by directly inhibiting tumor growth, probably through disrupting cell cycle progression, thus increasing cancer cell apoptosis. Meanwhile, the relatively low molecular weight of EgKI-1 (< 10 kDa) may allow it to penetrate tumor tissues effectively, thus facilitating its interaction with malignant cells. Therefore, EgKI-1 seems to be a promising therapeutic molecule against cancer, which may be considered in future treatment development.

### Echinococcal Antigens as Cancer Vaccine for Immunotherapy

Successful ICIs treatment depends not only on the expression of checkpoint protein but also the immunogenicity of the cancer cells, i.e., whether the tumor is “immune competent” or “immune deficient” (Kyi and Postow, 2016). “Immune competent” means that the immunogenicity of cancer cell is strong enough to induce immune response of infiltrating T cells, which are unable to function normally due to checkpoint blockade or other immunoregulatory mechanisms (Mougiakakos et al., 2010; Shields et al., 2010); in direct contrast, “immune deficient” means that the immunogenicity of cancer cell is not sufficient to be recognized, thus enabling tumor to escape from host immune surveillance even after the checkpoint blockade has been relieved by ICIs (Hanahan and Weinberg, 2011; Tumeh et al., 2014).

After near 40 years of extensive research with only limited success, it is now believed that cancer vaccine may have the potential to convert immune-deficient tumor amenable to ICIs treatment through activation of T cell immunity (Lippitz, 2013; Maeng and Berzofsky, 2019). In this context, an ideal cancer vaccine should be, first, able to stimulate strong Th-1 response specifically (since Th-2 response may facilitate cancer progression and metastasis), and second, tumor-specific (inducing adaptive immunity specifically against cancer cells while sparing normal cells). Many tumor-specific antigens have been identified and exploited. Most of them, however, cannot trigger proper and effective immune response (Buonaguro et al., 2010), including the tumor associated mucin-type O-glycans Tn, sTn, and T antigen (Fu et al., 2016), which are almost exclusively found in cancer cells and have been widely employed in cancer diagnosis and prognosis prediction (Kudelka et al., 2015). Notably, an sTn-based vaccine failed to benefit women with metastatic breast cancer despite high level of specific immunoglobulin G production and immunoglobulin M-to-immunoglobulin G seroconversion in a phase III multicenter study (Miles et al., 2011). A Th-2 dominant response may be an explanation for the failure of this vaccine, thus highlighting the importance of a Th-1-dominant response.

As noted above, the mucin-type O-glycans have already been detected both in adult and larval stage of *E. granulosus* and in patients with CE (Alvarez Errico et al., 2001; Casaravilla et al., 2003). On the other hand, accumulating evidences have demonstrated that hydatic cyst antigens can induce anticancer effect against several cancer types *in vitro* and in murine models (Aref et al., 2013; Darani et al., 2016; Rostami et al., 2018; Darani et al., 2018). Furthermore, immunization of mice with echinococcal antigens, whether Tn-like peptide or crude hydatid cyst antigens, resulted in high level of IFN-γ production, indicating a Th-1-polarized response (Noya et al., 2013). Taken together, these findings attempt us to hypothesize that the hydatid cyst antigens, especially the mucin like O-glycans, may induce anticancer effect through activation of Th-1-polarized immune response. Therefore, the mucin-like O-glycans of *E. granulosus* may be considered as potential candidate in future vaccine design. Since there are great differences between human and murine immune system, additional studies are required to verify this hypothesis.

## Conclusion

Accumulating evidences have suggested that the canine tapeworm *E. granulosus* can induce anticancer effect against several cancer types *in vitro* and in murine model, presumably through activating Th-1-polarized immune response with common antigens, especially the mucin-type O-glycans, and secreting molecules with anticancer potential, EgKI-1 in particular. Hopefully, these findings may provide some new insights into immunotherapy and replenish our arsenal against cancer. With a new era of immunotherapy at dawn, further studies are required to shed further lights on the mechanism underling the Th-1 response induced by mucin-type O-glycans and to identify additional ES products with anticancer potential. In addition to anticancer effect, several research groups have reported that *E. granulosus* could not only provide protective effect against *Taenia multiceps* infection in sheep (Gauci et al., 2008) but also against experimental colitis (Soufli et al., 2015; Khelifi et al., 2017) and airway inflammation (Wang et al., 2014; Kim et al, 2019). The exact mechanism underlying these protective effects remain largely unknown, possibly related to antigen similarity and immunomodulatory effect from echinococcal molecules, which are also worthy of further exploring.

## Author Contributions

GW and WX drafted this manuscript. GW designed the artwork and revised the manuscript according to reviewers’ comments. LS and ZX refined English writing. WX, ZX, and ZJ reviewed this manuscript.

## Funding

The publication of this review has been funded by A Major Infectious Disease Prevention and Control of the National Science and Technique Major Project (NO. 2018ZX10715004), Project from Department of Education of Sichuan (NO. 18ZB0241) and Research Project from Sichuan Health and Family Planning Commission (NO.16PJ511 ).

## Conflict of Interest

The authors declare that the research was conducted in the absence of any commercial or financial relationships that could be construed as a potential conflict of interest.
